# 40-Hz Binaural beats enhance training to mitigate the attentional blink

**DOI:** 10.1038/s41598-020-63980-y

**Published:** 2020-04-24

**Authors:** Bernhard Ross, Marc Danzell Lopez

**Affiliations:** 1 Rotman Research Institute, Baycrest Centre for Geriatric Care, Toronto, Ontario M6A 2E1 Canada; 2 Department of Medical Biophysics, University of Toronto, Toronto, ON M5G 2M9 Canada; 3 Faculty of Arts and Sciences, University of Toronto, Scarborough, ON M1C 1A4 Canada

**Keywords:** Attention, Consolidation

## Abstract

This study investigated whether binaural beat stimulation could accelerate the training outcome in an attentional blink (AB) task. The AB refers to the lapse in detecting a target T2 in rapid serial visual presentation (RSVP) after the identification of a preceding target T1. Binaural beats (BB) are assumed to entrain neural oscillations and support cognitive function. Participants were assigned into two groups and presented with BB sounds while performing the AB task on three subsequent days in a cross-over design. Group A was presented with 40-Hz BB during the first day and 16 Hz during the second day, while the order of beat frequencies was reversed in Group B. No sound was presented on the third day. MEG recordings confirmed a strong entrainment of gamma oscillations during 40-Hz BB stimulation and smaller gamma entrainment with 16-Hz BB. The rhythm of the visual stimulation elicited 10-Hz oscillations in occipital MEG sensors which were of similar magnitude for both BB frequencies. The AB performance did not increase within a session. However, participants improved between sessions, with overall improvement equal in both groups. Group A improved more after the first day than the second day. In contrast, group B gained more from the 40 Hz stimulation on the second day than from 16-Hz stimulation on the first day. Taken together, 40-Hz BB stimulation during training accelerates the training outcome. The improvement becomes evident not immediately, but after consolidation during sleep. Therefore, auditory beats stimulation is a promising method of non-invasive brain stimulation for enhancing training and learning which is well-suited to rehabilitation training.

## Introduction

Perceptual and motor skills can improve to a certain degree through training. However, successful training requires time and effort. Notably, the outcome of rehabilitation training could benefit from new methods for improving the efficacy of learning. One proposed method to accelerate learning employs non-invasive brain stimulation, paired with a training task^1^. The current study used the attentional blink paradigm to investigate whether auditory stimulation with binaural beats during the training improves the performance in detecting a target within a rapidly presented sequence of visual stimuli.

Non-invasive brain stimulation using transcranial magnetic stimulation (TMS) and transcranial direct current stimulation (tDCS) has been shown to improve learning and training outcomes^2,3^. However, the underlying mechanisms are not well understood. It has been suggested that the electric or magnetic stimulation modulates the neural membrane potential^4^ and causes inhibitory or excitatory effects depending on stimulus parameters^5^. Also, the stimulation may increase synaptic efficacy through stimulus-induced long-term potentiation^6^ and thus facilitates neural plasticity underlying learning. A recent approach to non-invasive brain stimulation employs the concept that rhythmic magnetic or electric stimulation could entrain neural oscillations in a specific frequency band, such as gamma oscillations^7,8^.

As an alternative to electrical stimulation, rhythmic sensory stimulation could entrain neural oscillations. EEG and MEG studies showed synchronization of brain activity at the frequency of stimulation and its harmonics in and beyond the corresponding sensory brain areas with visual flicker^9,10^, somatosensory tactile stimulation^11,12^ and auditory rhythm^13,14^. Specifically, binaural beats (BB) have been proposed as a particularly beneficial stimulus sound because they involve a complex interaction between brain processes. Binaural beats occur when both ears are presented with pure tones of slightly different frequencies^15^. The frequency difference causes a periodic change in the interaural phase difference. Neurons in the brainstem are sensitive to such interaural phase differences and generate the BB^16^. Binaural integration at the cortical level leads to the perception of a sound with a single pitch corresponding to the mean of both tones with modulation of the amplitude at a rate equal to the difference between the two tonal frequencies^17^.

Brain oscillations entrained by the BB have been recorded with EEG and MEG similar to the responses elicited by amplitude-modulated sounds at theta frequencies^18,19^ and predominantly at gamma frequencies around 40 Hz^13,19,20^, although the beat salience is weaker at 40 Hz compared to low-frequency beats^17^. Entrainment of gamma oscillations is of specific interest because of the role of gamma oscillations for attention^21^, feature binding^22,23^, memory^24^, and learning^25^. Beneficial effects of BB stimulation have been reported for memory^26–29^, attention^30^, creativity^31^, anxiety control^32^, modulation of mood states^27,33^, and pain perception^34^.

We studied the effect of concurrent binaural beat stimulation on training to remediate the attentional blink (AB) effect. The AB refers to the lapse in perception of a target T2 in a rapid serial visual presentation (RSVP) task during a short time interval following the identification of a target T1^35,36^. The AB effect has been explained as limits in the temporal dynamics of focusing attention^37^, competition within the limits of a visual short term memory^38^ or limited capacity for later decision making^39^. Those limitations may be structural, and therefore, the effects of training to overcome the AB effect may be marginal. However, the effects of intense training over multiple days have been reported^40,41^. Seemingly, a period of sleep between sessions plays an essential role in the effect of training the AB^42^. The hypothesis that auditory beats interact with the visual AB stimuli requires synergetic interaction between auditory and visual perception. Supporting evidence for non-conflicting cross-modal interaction came from a study that showed that auditory processing was improved when participants were involved simultaneously in a visual and auditory AB task^43^.

The specific aim of this study was to investigate whether a 40-Hz gamma-band BB stimulation had a stronger effect on training the AB performance than a BB stimulation outside the gamma band. While previous studies compared groups of participants, exposed with different BB frequencies^44^, we employed a crossover protocol providing better statistical power to show treatment effects. Participants in the current study received a moderate amount of training the AB paradigm within three sessions on subsequent days. During the first two days, participants were exposed to BB stimulation at different frequencies suitable to strongly or weakly entrain neural gamma oscillations. The research question was whether the stimulation with different BB frequencies would affect the time courses of AB performance improvement. Half of the participants were presented with 40-Hz BB during training on the first day and 16-Hz on the second day, while the order of BB frequency was switched for the other half of the participants. No sound stimulation was provided on the third day. This design allowed for analyzing performance changes on the AB task between groups but also the effect of BB treatment as a within-group variable.

## Methods

### Participants

Twenty-nine young, healthy volunteers, recruited through the Rotman Research Institute volunteer database and the local community, completed the study. One participant was excluded as an outlier because they failed to perform the task; another was excluded for technical reasons. The participants were randomly assigned to two treatment groups. Seven women and seven men with the mean age of 21.8 years (range, 18 to 28 yrs, std = 3.3 yrs) were assigned to group A (n = 14). Group B (n = 13) consisted of eight women and five men with a mean age of 21.9 years (range, 18 to 32 yrs, std = 3.6 yrs). All participants had normal or corrected-to-normal vision and normal hearing. They were self reportedly healthy without a history of neurological or psychiatric disorders and free of medications. All participants were naïve to the type of visual experiment used and did not have experience with binaural beat stimulation. Participants provided their informed consent for participating after receiving full information about the nature of the study. The study protocol was prepared in accordance with the principles of ethical research as of the declaration of Helsinki^45^ and was approved by the Research Ethics Board at Baycrest Centre for Geriatric Care (REB 17–22).

### RSVP stimulus sequence

**Figure 1:**
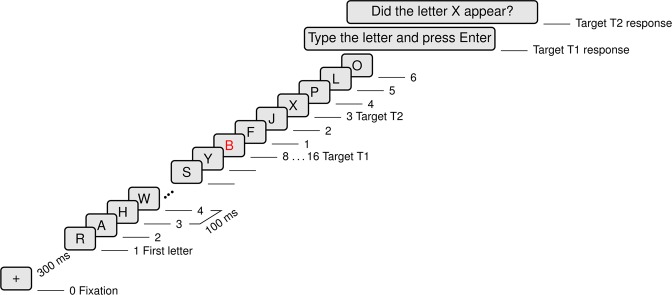
Rapid serial visual presentation (RSVP) for the attentional blink (AB) experiment.

The stimulus sequence was a rapid serial visual presentation (RSVP) of the 26 capital letters of the alphabet in random order without repetition of a letter. The stimuli were Helvetica font letters of 10 mm height in the centre of a 15″ LCD screen, placed at 60 cm viewing distance from the participant. The letters were presented for 33 ms followed by a blank-screen inter-stimulus interval of 67 ms, which resulted in the presentation rate of ten letters per second. The RSVP sequence (Fig. 1) started with a fixation cross 300 ms before the sequence of black letters was presented on a light gray background. The T1 target was a random letter which appeared in red colour between the 8^th^ and 16^th^ position in the sequence. The T2 target was the letter X without colour emphasis. In half of the trials, the six-letter sequence directly following T1 contained T2. The other half of the trials did not contain T2. In total, the sequences contained 14 to 22 letters following the initial fixation cross and were of durations between 1.7 s and 2.5 s. After the RSVP sequence, participants were asked to type the T1 letter and respond to whether they detected the T2 target using the arrow keys on a computer keyboard. There was no time limitation for the response and the next sequence started 1.0 s after the T2 response. The stimulus presentation was controlled by Presentation software (Neurobehavioral Systems, Berkeley, CA).

### Binaural beats

Pure tones of 420 Hz and 460 Hz presented to the left and right ear, respectively, were perceived as a 40-Hz BB with a 440 Hz fundamental tone. Pure tones of 431.85 Hz and 448.15 Hz were used to produce the 16-Hz BB with the same fundamental tone. Sound files of 15 min duration were created with Matlab and presented continuously in the background during the RSVP experiment. The sound intensity was set to 60 dB sound-pressure level, equivalent to 48 dB normal hearing level. The sound intensity was controlled by a clinical audiometer (GSI 61, Grason-Stadler, Eden Prairie, MN), and the sounds were presented with insert phones (EAR 3 A, Etymotic Research, Elk Grove Village, IL). The experiments were performed in a soundproof booth.

### Study design

All participants performed the RSVP paradigm on a series of three days according to their convenience. Most participants completed the study within three consecutive days; the longest interval between the first and last session was seven days. At each session, the participants performed four experimental blocks. Each experimental block consisted of ten trials for each of the six lag positions between T1 and T2 resulting in a total of 60 RSVP trials per block. Each block began with a brief instruction and six practice trials. The first block in each session was a control task without the red coloured T1 target, in which participants detected the letter X, later used as the target T2. During the three subsequent blocks, participants identified the target T1 and detected T2. About 10 to 12 minutes of time was required for each block. Participants were instructed to take a short break between the blocks to mitigate the possible effects of fatigue. A session was completed within less than one hour. The participants were randomly assigned to two groups which differed according to the order of the BB stimulation. Group A listened to 40 Hz beats on the first day and to 16 Hz beats on the second day. Group B listened first to 16 Hz beats, and the 40 Hz beats in the second session. No BB stimuli were presented on the third day for either group.

### MEG recordings

In a control group of n = 5 healthy young adults, we recorded brain responses with MEG to investigate the entrainment of oscillatory responses by the auditory and visual stimuli. In two sessions of about one hour MEG recording each, the participants were presented with the RSVP sequences and listened to the BB sounds at 16.3 Hz and 40 Hz, respectively. In a third one-hour MEG session, the participants listened to the BB sounds only. The RSVP stimuli were twenty alphabetical letters, presented in random order with an inter-stimulus interval of 100 ms without the AB task. The BB sounds were presented in short bursts of 3.0 s duration strictly phase-locked to the visual stimuli. In contrast, the BB sound had been continuously presented for the behavioural AB task, and the phase relation between auditory and visual stimuli varied between trials. The strict phase-locking between the stimuli during the MEG recording allowed for analyzing putative interactions between the visual and auditory stimuli. For each participant, we recorded a total of 980 trials with RSVP stimulation and BB of 16.3 Hz and 40 Hz, respectively, and 480 trials each with sole BB stimulation at 16.3 Hz and 40 Hz. The MEG was recorded with a 151 channel axial gradiometer-type whole head MEG system (CTF MEG, Coquitlam, BC, Canada) at the Rotman Research Institute. Details about the MEG recording and data pre-processing can be found in our previous reports^13^. The MEG data analysis aimed at comparing the entrainment of auditory responses at 16.3 Hz and 40 Hz and exploring whether 10-Hz oscillations, elicited by the rhythm of the visual stimuli, were different when the participants were concurrently presented with BB sounds at 16.3 Hz or 40 Hz. The data analysis was performed in the MEG sensor domain of the magnetic field signals. Outcome measure of the analysis was the inter-trial phase coherence^46,47^ obtained from FFT spectrum analyses for the BB responses and time-frequency analysis based on Morelet wavelets^48^ for the RSVP responses. The phase coherence indicates the degree of synchronization regardless of the absolute signal amplitude and thus allows of fair comparison between spectral components at different frequencies.

### Analysis of behavioural data

Accuracy measures for the AB task were calculated separately for each lag (one to six) as the difference between the hit rate and false-alarm rate of T2 detection considering only responses following correct identification of T1. Measuring the AB effect and its training-related modulation requires comparing the accuracy at a short lag versus accuracy at a long lag^49^. Previous studies using similar RSVP stimulation as in our study showed a most pronounced AB effect at the lag of two or three (180 to 270 ms)^35,36,50^ and specific effects of training were found for the target at a lag of two^51–53^. Therefore, we observed the T2 | T1 accuracy at a lag of two and compared this measure with the accuracy in the control condition, assuming that the accuracy in the control condition reflects the asymptotic T2 | T1 accuracy for a long lag. The data were analyzed with ANOVA and t-tests using the ez-anova package of R^54^. The data were tested for sphericity using Mauchly’s test and Greenhouse-Geisser correction was applied when necessary. The P-values of posthoc comparisons were corrected for multiple comparisons using the Holm-Bonferroni method.

## Results

### Entrainment of brain responses

**Figure 2:**
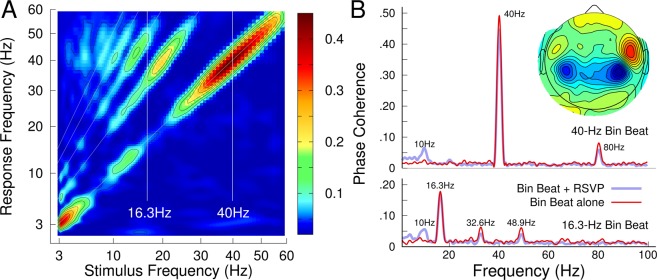
Binaural beat entrained brain oscillations. (**A)** Frequency spectra of auditory cortex responses to beat stimulation between 3 Hz and 60 Hz^13^. The beat frequencies of 40 Hz and 16.3 Hz were chosen for experimental conditions with strong and weaker entrainment of 40-Hz oscillations, respectively. (**B**) Spectra of MEG brain responses to 40 Hz and 16.3 Hz binaural beat stimulation. The spectra were obtained as the group mean of the maximally responding sensor above the right temporal lobe with auditory beat stimulation alone and in combination with the visual RSVP stimulation are overlaid. The topographic map of the MEG responses at 40 Hz in an individual participant reveals dipolar magnetic field patterns above bilateral temporal lobes, the location of auditory cortices.

One crucial design factor for the study was the choice of BB frequencies to provide a stark contrast between strong and weaker entrainment of gamma oscillations. The choice of BB frequencies was informed by previous MEG research. Previously, we studied how the auditory cortex responds to beat stimuli at various rates between 3 Hz and 60 Hz^13^. The brain responded preferentially with oscillations at 40 Hz (Fig. 2A). Notably, a stimulus at subharmonics of 40 Hz, i.e., at 20 Hz, 13.3 Hz, 10 Hz, etc., elicited a prominent response at 40 Hz. Thus, interpreting a contrast between beat stimulation at alpha (10 Hz), beta (20 Hz) and gamma (40 Hz) could be challenging because all beat frequencies would elicit brain responses at 40 Hz. Specifically, a beat stimulation at 20 Hz would not elicit a noticeable response at 20 Hz but a strong response at 40 Hz. Therefore, we chose the beat frequencies of 40 Hz and 16.3 Hz, the geometric mean of 20 Hz and 13.3 Hz, for a strong and weaker 40-Hz response, respectively. Figure 2A demonstrates that the auditory cortex activity barely synchronizes to the stimulus at 16.3 Hz and does not generate oscillations in the 40-Hz gamma frequency range.

The phase-coherence spectra of MEG recorded binaural beat responses in Fig. 2B predominantly show synchronous activity at 40 Hz and a weaker contribution at the first harmonic at 80 Hz when the beat frequency was 40 Hz. In contrast, the 16.3 Hz beat elicited a much smaller response at the beat frequency and even smaller contributions in the gamma band at 32.6 Hz and 48.9 Hz, two and three times the beat frequency. The MEG recordings confirmed that the experimental conditions constituted a contrast between strong and weaker entrainment of gamma oscillations when the beat frequencies were 40 Hz and 16.3 Hz, respectively. Figure 2B also illustrates that concurrent visual stimulation at the 10-Hz rate did not significantly affect the magnitudes of the auditory response. The topographic map of the 40-Hz response (Fig. 2B) shows dipolar patterns above bilateral temporal lobes, the origin of the auditory cortex, suggesting predominant contributions from auditory cortex activity. In contrast to the magnetic field topography of a response elicited by an amplitude-modulated sound, the binaural beat response was of almost opposite polarity in left and right auditory cortices consistent with earlier reports about binaural beat and AM responses^20^.

**Figure 3:**
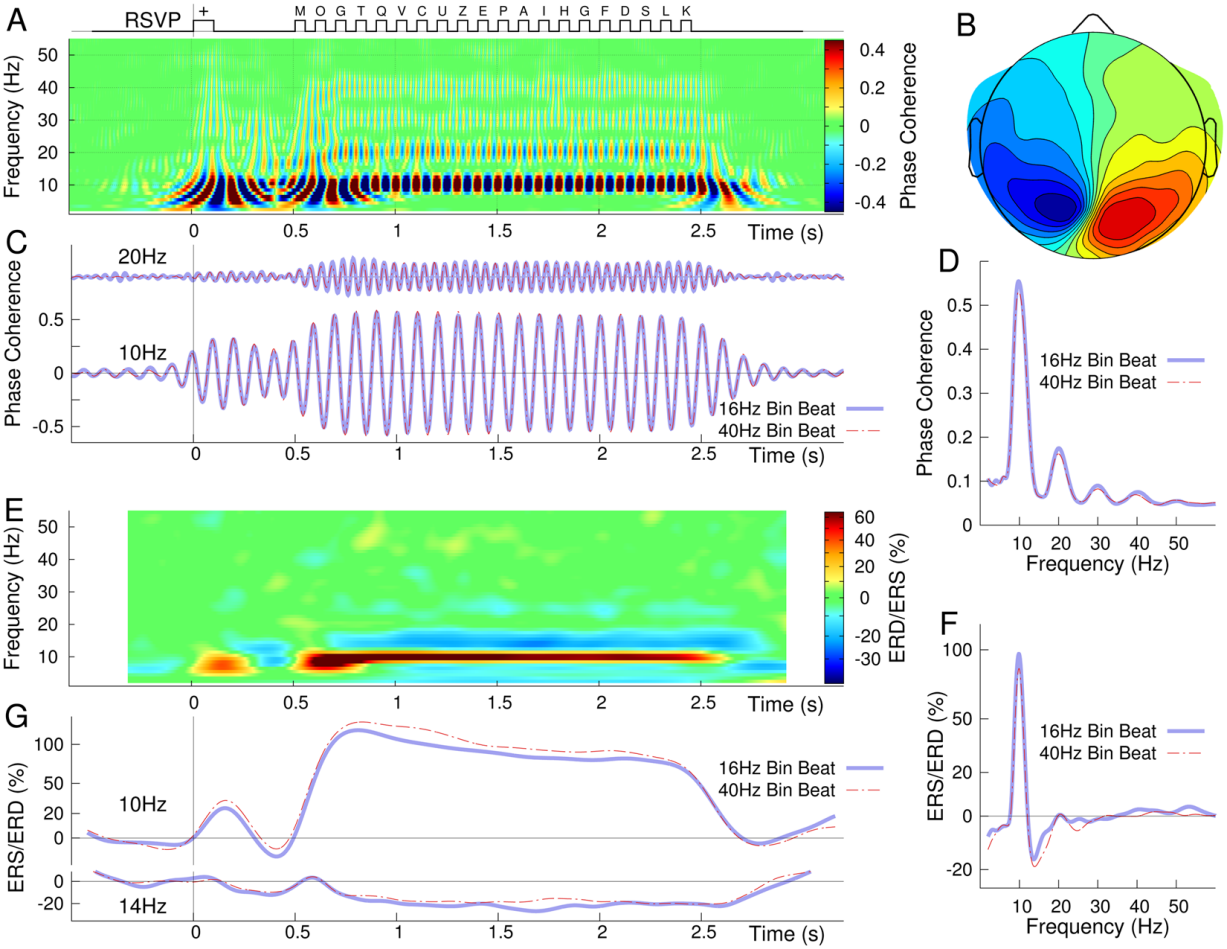
Entrainment of alpha oscillations through the rapid visual stimulation. (**A**) Time-frequency map of the real part of phase coherence indicates strong synchronization of 10 Hz oscillations with the rhythm of the visual stimuli (top) as well as weaker synchronizations at multiples of 10 Hz during the time interval of RSVP stimulation. The time-frequency map was obtained as group mean across the maximally responding occipital sensors in n=5 individuals. (**B**) The topographic map of 10 Hz oscillations shows a dipolar pattern above the occipital lobe in an individual participant. (**C**) Group mean time series of oscillations at 10 Hz and 20 Hz. The responses obtained during beat stimulation at 40 Hz and 16.3 Hz overlay almost perfectly. (**D**) Spectrum of oscillations phase-locked with the stimulation rate of 10 Hz and its harmonics. (**E**) Time-frequency maps of event-related synchronization (ERS, power increase relative to baseline) and desynchronization (ERD). The visual stimuli induce a power increase at 10 Hz of similar magnitude for the concurrent beat stimulation at 16.3 Hz and 40 Hz. The 10-Hz power increase is accompanied by a stimulus related power decrease in the upper alpha band. (**F**) Frequency spectrum of induced power changes, showing power increase at 10 Hz and decrease at 14 Hz. (**G**) Time series of ERS and ERD at 10 Hz and 14 Hz.

The time-frequency map of phase synchronous responses to the visual stimulation shows transient responses to the onset of the visual cue, the onset of the letter sequence, and the ending of the stimulus sequence and most prominently continuous 10-Hz oscillations during presentation of the letter sequence (Fig. 3A). Moreover, the time-frequency map indicates entrained oscillations at 20 Hz but less expressed in the gamma band at 30 Hz and 40 Hz. The topographic map (Fig. 3B) shows a dipolar pattern of the 10-Hz oscillations above the occipital brain. Time series of 10-Hz and 20-Hz activity show steady oscillations during the visual stimulation (Fig. 3C). The frequency spectrum, obtained during the 0.5 s to 2.5 s time interval of periodic visual stimulation, shows the prominent peak at the 10-Hz stimulation rate and smaller peaks at harmonic frequencies (Fig. 3D). Both the time series and the frequency spectra are overlaid for the responses with concurrent 40-Hz and 16 Hz binaural beat stimulation and did not exhibit differences between the beat frequencies. The time-frequency map of the event-related changes in signal power (ERS/ERD) shows a strong power increase during the visual stimulation, accompanied by a power decrease in the upper alpha band (Fig. 3E). The time series for 10-Hz ERD and 14-Hz ERS and the ERS/ERD spectra obtained with concurrent auditory stimulation at 40 Hz and 16 Hz were overlaid in Fig. 3F,G and showed closely similar characteristics.

In brief summary, the MEG recordings demonstrated entrainment of multiple types of brain oscillations. The visual stimulation entrained 10-Hz oscillations above occipital areas, while the concurrent binaural beat stimulus elicited strong gamma responses at the 40-Hz beat rate and substantially weaker gamma oscillations when the beat rate was 16.3 Hz.

### Attentional blink performance

**Figure 4:**
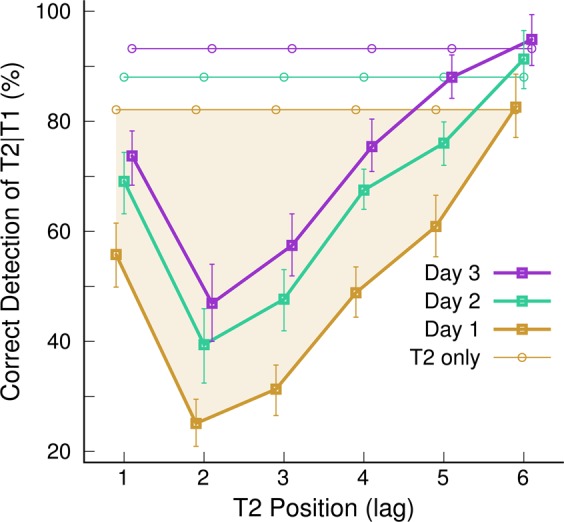
Grand mean attentional Blink effect, indicated by the percentage of correctly detected T2 letters conditional the correct detection of T1 at the three days. The error bars denote the 95% confidence limits of the group mean. The horizontal lines depict the detection rate in the control experiment without detecting the target T1. The shaded areas indicates for the first day the AB effect referenced to the accuracy in the control condition.

The group mean accuracy measures for T2 | T1 detection, visualized for the three sessions in Fig. 4, revealed the characteristic AB effect of impaired accuracy for detecting the target T2 during the 500-ms interval following T1. The accuracy was measured as the percentage of detected T2 targets conditional to the correct identification of the preceding T1 target minus the percentage of false alarms. The ANOVA for the detection accuracy revealed an effect of the T2 position (F(5,130)=65.4, P < 0.0001, η^2^ = 0.72), an effect of sessions (F(2,52)=100, P < 0.0001, η^2^ = 0.79), and an interaction between sessions and the T2 position (F(10,260)=3.06, P = 0.0011) because the performance increase was depending on the T2 position. The group-mean accuracy was lowest at the lag of two. Pairwise comparisons between the accuracy at lag of two with all other lags found a Holm-Bonferroni corrected P-value of 0.03 for lag of three and p < 0.0001 for all other lags (t(130)=8.65, 2.47, 8.0, 11.26, 15.64 for lag of 1, 3, 4, 5, and 6, respectively). Accuracy increased between first and second day (t(52)=8.92, P < 0.0001) and between the second and third day (t(52)=7.74, P < 0.0001). Also, the performance in the control condition, which required only detecting T2 without identifying T1, increased between sessions (F(2,52)=26.9, P < 0.0001, η^2^ = 0.51). However, showing a general improvement in accuracy across all lags is not sufficient to conclude a modulation of the AB effect^49^. Therefore, for subsequent analyses, the AB effect was measured as the difference between accuracies for the T2 | T1 detection in the AB task and the control condition as

$$AB\_effect=p{(T2|T1)}_{ABtask}+100 \% -p{(T2)}_{controltask}.$$

The AB effect, according to this definition, is depicted by the shaded area in Fig. 4 for the group-mean data on the first day.

**Figure 5:**
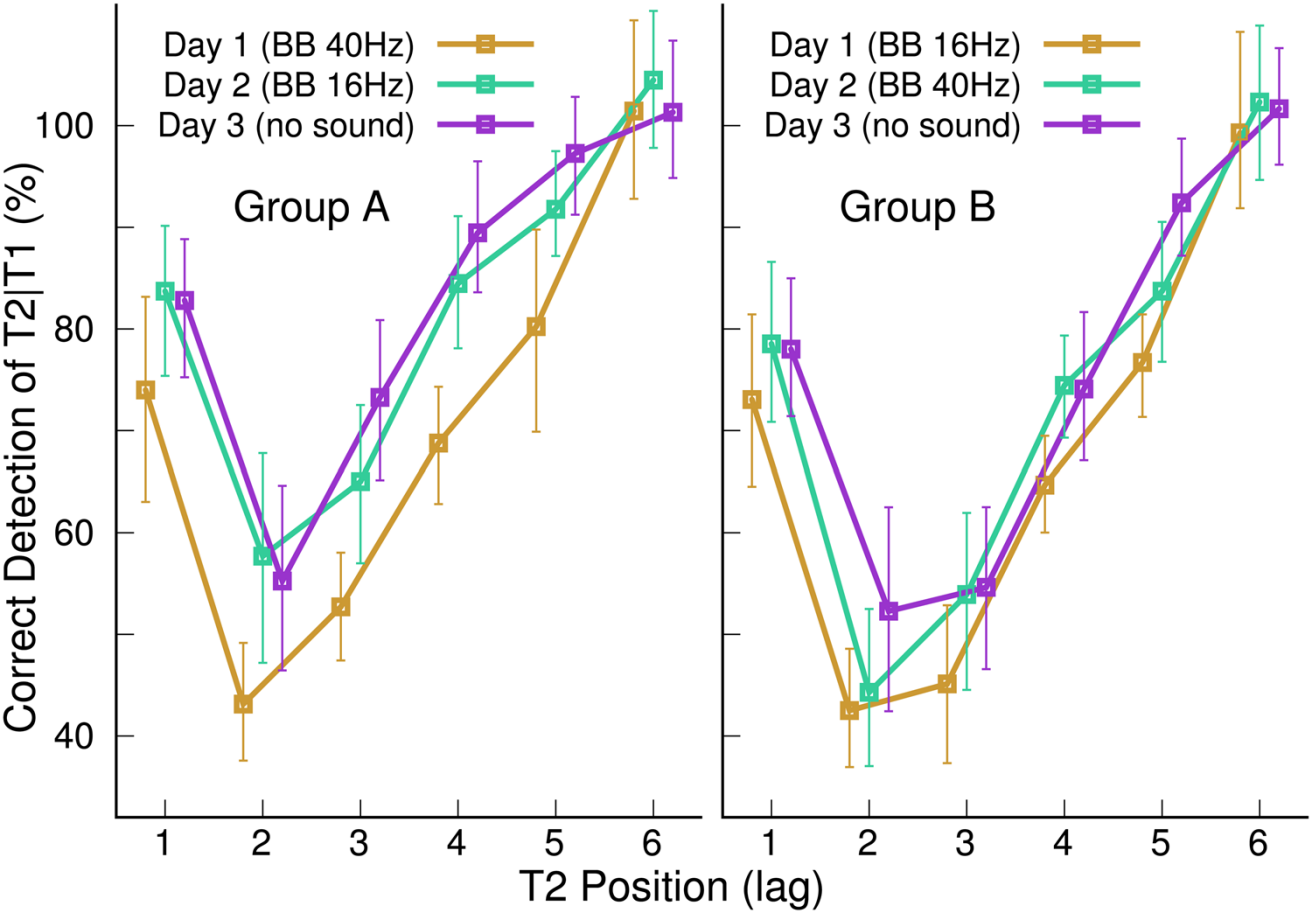
Attentional Blink effect indicated by the mean performance at the three days in both experimental groups.

The resulting group mean AB characteristics are shown in Fig. 5 separately for the two treatment groups. It becomes clear that the improvement in the control task accounted for most of the training effects since the distances between the graphs for the different days in Fig. 5 are smaller compared to Fig. 4. Nonetheless, Fig. 5 provides the first hint of differences in the trajectories of training effects between the groups. For example, participants in group A seemed to improve after the first day with 40-Hz BB stimulation, whereas lesser improvement occurred after the second day with 16-Hz BB. In contrast, participants in group B seem to improve little after the first day of training under 16-Hz BB stimulation. Specifically, no difference was shown between the first and second day for the AB at a lag of two. However, the performance seems to increase after the second day of training with 40-Hz BB. We focused the further analysis on the AB at a lag of two, for which previous studies showed an effect of training.

**Figure 6:**
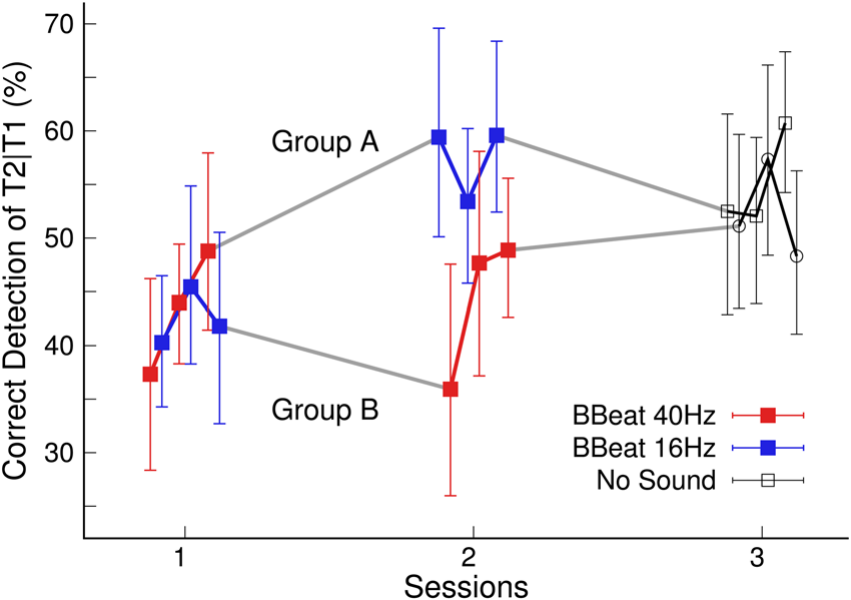
AB effect during the three experimental blocks per day and three subsequent days. Group A was exposed to 40 Hz binaural beats at the first day and 16 Hz at the second day. The order of binaural beat stimulation was reversed in group B. No sound stimulation was applied at the third day.

Figure 6 shows how the group mean accuracy for T2|T1 detection developed for all experimental blocks within a session and between the three subsequent sessions. An ANOVA with between-group factor ‘treatment group’ and within-group factors ‘session’ and ‘block’ revealed a main effect of ‘session’ (F(2,48) = 8.0, P = 0.001, η^2^ = 0.03). Pairwise comparisons indicated an overall performance gain between the first and last session (t(48) = 3.83, P = 0.0011). However, the effects of ‘block’ or interactions between ‘block’ and ‘session’ or ‘group’ were not significant (F < 2.0 for all). Thus, this step of the data analysis did not provide evidence for a training effect within a session, with neither 40-Hz nor 16-Hz BB stimulation. Therefore, for further analysis, the accuracy measures for individual blocks were lumped together for each session, and we analyzed how the AB performance changed between sessions.

**Figure 7:**
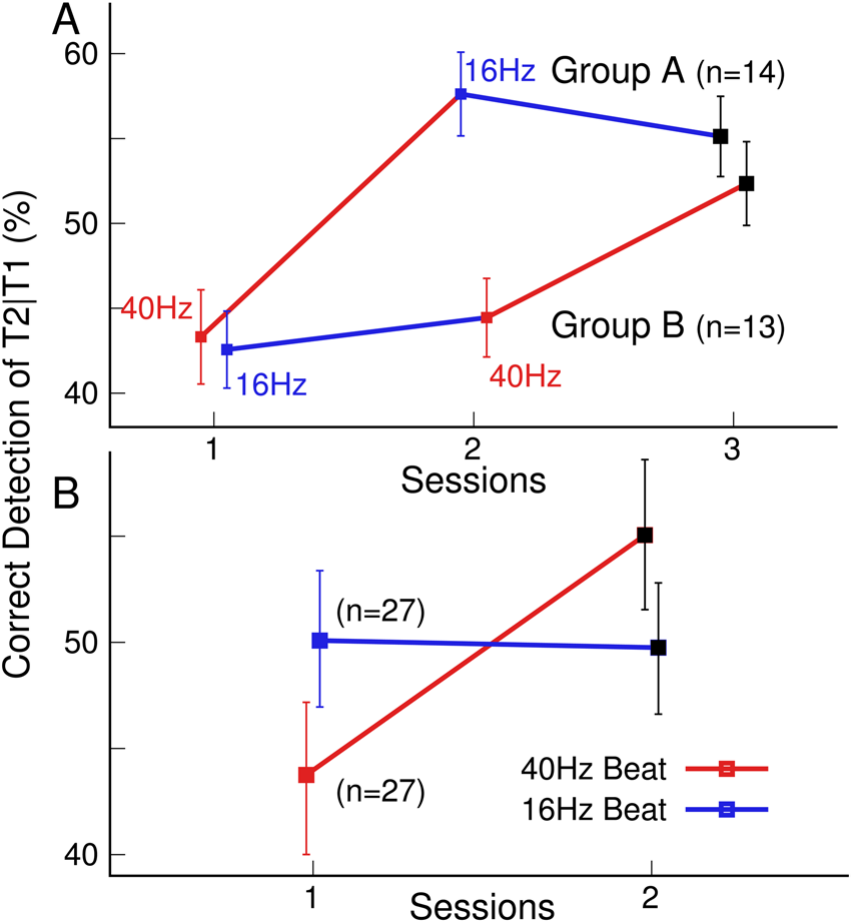
AB effect under BB stimulation at 40 Hz and 16 Hz. (**A**) Trajectories of AB performance across the three sessions for both experimental groups. (**B**) AB performance along the treatment arms with 40-Hz and 16-Hz binaural beat stimulation. The graphs show the change in AB performance between a session with 40-Hz or 16-Hz BB stimulation and the following session.

Figure 7A shows the trajectories of the group mean AB effects, averaged across the three repeated blocks for each of the three sessions and for both groups. Both groups improved their performance between the first and last sessions (group A: t(13 = 2.61, P = 0.022, group B: t(12) = 2.36, P = 0.036). The overall gain also became evident through the significant paired t-test for the combined groups (t(26) = 3.58, P = 0.0014). Participants in group A improved between the first and second sessions (t(13) = 3.05, P = 0.009), but not between the second and third sessions (t(13) = −0.63, P = 0.54). In contrast, participants in group B did not improve between the first and second sessions (t(12) = 0.49, P = 0.63) but showed a tendency for improvement between the second and third sessions (t(12) = 1.88, P = 0.085). Unpaired t-tests did not show group differences in the first session (t(25) = 0.21, P = 0.84) and the third session (t(25) = 0.81, P = 0.42) but a significant group difference at the time of the second session (t(25) = 3.88, P = 0.0007). Thus, both groups who performed the same AB tasks and received the same amounts of auditory stimulation were not different at the beginning and the end of the experiment. However, the trajectories diverged at mid-point of the experiment according to different orders of stimulation with different BB frequencies.

The final step of the data analysis compared the performance change along the different treatment arms, as illustrated in Fig. 7B. Here, the accuracy measures in both groups were combined according to the frequency of BB stimulation applied during that session. The combined accuracy measures were compared between the session with BB stimulation at each frequency and the subsequent session. The two-way ANOVA with the within-group factors ‘treatment’ (40-Hz BB vs. 16-Hz BB) and ‘session’ revealed a main effect of ‘session’ (F(1,26) = 12,8, P = 0.0014, η^2^ = 0.33), no mean difference between treatments (F(1,26) = 0.11, P = 0.74) but a ‘treatment’ × ‘session’ interaction (F(1,26) = 5.2, P = 0.031). The performance gain was significant after 40-Hz BB stimulation (t(26) = 3.54, p = 0.0015) but not after 16-Hz BB stimulation (t(26) = 0.39, P = 0.70).

The ANOVA for the T1 performance showed an effect of ‘sessions’ (F(2,50) = 14.3, P < 0.0001). T1 accuracy improved between the first and second sessions (t(26) = 2.19, P = 0.036) and between the second and third sessions (t(26) = 3.24, P = 0.004). There was no effect of ‘lag’ (F(5,125) = 0.7); specifically, the T1 performance was not affected at a lag of one when T2 immediately followed T1. Also, no effect of ‘group’ or a ‘group’ × ‘session’ interaction was found.

## Discussion

Over the time course of practicing the AB task in three sessions, all participants improved their performance. While no performance gain was observed within a session, the performance enhanced between sessions at subsequent days after a night of sleep. Behavioural performance was not different during sessions with 16 Hz or 40 Hz BB stimulation. Most notably, the between-sessions performance gain was largest after a session with 40-Hz BB stimulation. The results demonstrate that 40-Hz BB stimulation concurrent with a training task enhances the efficacy of the training.

Previous findings of a most pronounced AB effect when the rhythm of the RSVP stimulation coincided with alpha and lower beta frequencies^55^ suggested that brain oscillations at alpha and beta frequencies may play a role in the neural mechanism underlying the AB. It has been shown that stronger suppression of alpha oscillation during anticipation of the T2 target decreased the T2|T1 accuracy in an AB task^56^. Moreover, visual perception was affected by the phase of alpha oscillations in relation to the time of the stimulus onset^57–59^. However, the explanation that the rhythm of the RSVP stimulation may entrain alpha oscillations, which in turn interacts with subsequent visual processing remains still to be confirmed through experimental studies^55^. One finding was that rhythmic auditory stimulation at 10-Hz entrained alpha oscillations and reduced the AB effect^60^. Another striking finding was that listening to music while performing the AB task improved the AB performance^61^. An explanation of such a paradoxical effect could be that listening to music attenuated alpha oscillations in visual areas^62^ and thus counteracted the alpha entrainment caused by the rhythmic visual stimulation. These findings support the hypothesis that entrained alpha oscillations underlay the AB. Our MEG recordings demonstrated strong entrainment of 10-Hz oscillations, which was also expressed by a substantial increase in the 10-Hz power compared to baseline. However, the signal power in the upper alpha band decreased during visual stimulus presentation. Thus, the stimuli induced alpha ERD and ERS simultaneously. Both types of event-related alpha power change have been interpreted as active processes^63^. However, further research is required before conclusively explaining the role of alpha oscillations for the AB effect.

In our current study, participants were exposed to continuous tones, which other than musical stimuli, did not contain temporal transients, and thus unlikely reset the level of alpha oscillations. On the other hand, the rhythm of the BB stimulation could have interacted with the rhythm of the RSVP stimulation. It has been shown that cross-modal brain areas respond to rhythmic visual and auditory stimulations with oscillations at intermodulation frequencies^64^. Such cross-modal interactions could have affected visual perception^65^ and attention^66^, and such effects would be frequency-specific, i.e., the cross-modal interactions would have been different for 16-Hz and 40-Hz BB stimulation. We compared the visually entrained MEG alpha oscillations when participants were concurrently presented with 40-Hz or 16-Hz beats and did not find significant effects on the 10-Hz oscillations and its harmonics. Moreover, we compared the auditory beat responses with and without concurrent visual stimulation and did not find evidence for interactions. Although entrained auditory and visual responses were strongly expressed in the response spectra, we did not find inter-modulation products. Cross-modal interactions potentially could have immediately affected the AB performance and could have resulted in differences in the AB effect during sessions with 16-Hz and 40-Hz BB stimulation. The MEG results of absent or minimal interactions between the primary sensory responses correspond to the behavioural results of no immediate effect of the BB stimulation.

Instead of an immediate effect of the BB stimulation, AB performance changes occurred with a delay between sessions. Such delayed effect is characteristic of perceptual learning requiring consolidation during sleep^67^. That sleep after training improves the AB performance has been shown previously^51^. Thus, we assume that the BB stimulation interacted with a neural network underlying learning and training rather than immediately with perception.

Systematic studies about which BB frequency would provide the most beneficial training effects have not been conducted so far. In most studies, likewise, in our study, the BB frequency was selected according to a hypothesis about the role of entrained neural oscillations. Our study was designed to induce a strong (40 Hz) or weaker (16 Hz) entrainment of gamma oscillations through BB stimulation. The focus on 40-Hz oscillations is in line with several reports about the positive effects of gamma-frequency BB. For example, 40-Hz BB improved working memory recall^27^, gamma-band BB impacted the control of visual feature binding^68^, and 40-Hz BB supported cognitive control of decision making^69^. Another study showed that 20-Hz BB improved long term memory while 5-Hz BB impaired memory compared to a white noise control condition^26^. However, the 20-Hz BB may have entrained oscillations in a 40-Hz gamma network.

One important finding in our study was that the AB performance improved between sessions but no gain was observed within a session with either type of BB stimulation. Previous studies about training effects on RSVP tasks involved massive training with hundreds of repetitions of the task, commonly over the time course of several days, thus including intervals of sleep^40,70^. Whether a period of sleep is required for a performance gain had been investigated in a study in which participants performed AB tasks in the morning and evening of the same day^51^. Only participants who took a nap between the sessions improved the detection performance for targets at lag of two, while participants who continued their daily activities did not gain in performance. Our finding of a performance gain between sessions, assuming periods of sleep in between, may be explained by a two-step effect of learning. Perceptual learning depends on improvements at the levels of sensation and perception as well as at the cognitive level of decision making. Recent human studies showed that improvements at the latter level of decision making might predominantly determine the learning outcome^71^. We speculate that the performance in the AB task depends on a top-down model of decision making. When repeatedly performing the AB task during the training, participants better understand and refine the rules underlying the model. However, the model will be updated only after consolidation over a night of sleep, and the updated model can be used on the following day. Thus, the effect of training becomes evident on the next day.

The observed effects of training on the AB task were robust enough to conclude significant differences between the treatment groups and significant differences between treatments with a strong or weaker gamma-frequency BB. However, the total effect of the training was small, and after the training, the participants were still far from overcoming the AB effect completely. Such incremental performance gain is consistent with previous studies showing larger effects only after massive training with hundreds of repetitions^40,41^. The current finding that presentation with 40-Hz BB resulted in larger gain than 16-Hz BB suggests that gamma-frequency BB could be of specific interest for improving rehabilitation training. The performance increments in rehabilitation training, e.g., after brain injury or a stroke, are commonly tiny. Sensory stimulation such as through auditory beats, could accelerate the training.

A recent study of the effects of BB on performance in the AB showed complete elimination of the AB during stimulation with 40-Hz BB but no difference between stimulation with a 10-Hz BB and a control condition without beating sound^44^. However, the elimination of the AB was observed only in a subgroup of participants with a low spontaneous eye-blink rate. Another study of inter-subject variability in AB performance^72^ reported an almost absent AB effect in individuals with low eye-blink rate even without BB stimulation. None of the participants in the current study performed at the ceiling.

A study of monaural beats found similar effects on anxiety, mood, and memory, as reported with binaural beats^73^. Thus, AM sounds could serve as rhythmic auditory stimuli. One advantage of using AM sounds is that presentation with a single loudspeaker would be feasible instead of wearing headphones. EEG and MEG studies showed similar effects of AM and BB stimuli on the auditory cortex responses^18,20,74^. However, we know little about entrainment of neural oscillations beyond the auditory cortex, which may differ for AM and BB sounds. Coincidence detectors in the auditory brainstem detect the BB induced rhythmic changes in the interaural phase difference and generate the BB response. This neural network is used for sound localization and may project to different cortical areas than the afferent projections for the AM sound. Our MEG results showed a magnetic-field topography distinctly different from an AM-sound response. An fMRI study found different pathways of activation for sound localization versus sound recognition^75^; specifically, sound localization involved bilateral inferior frontal and parietal lobes. Future studies still have to show whether BB stimuli provide better entrainment of neural oscillations beyond primary sensory areas than AM sounds would do.

Continuous stimulation with BB sounds could be more acceptable than AM sounds because the salience of BB is low, specifically at 40 Hz. In contrast, the buzzing AM sound could be perceived as annoying by some listeners. Also, low-intensity tones can induce the BB. The BB can become audible even when the sound intensity in one ear is up to 20 dB below sensation threshold^76^. Also, BB has been created from a wide-band noise^77^ and has been embedded in music^32^. Using such BB sounds could enhance compliance with listening to the stimuli. However, the efficacy of music, noise, or subliminal sounds for non-invasive brain stimulation has yet to be proven.

In summary, this study provided the first evidence for the beneficial effects of BB stimulation on the trajectory of training to overcome the AB effect. Common with electric or magnetic non-invasive brain stimulation, the underlying mechanism requires further research. However, rhythmic sensory stimulation could be considered as an efficient alternative or complement to currently used non-invasive brain stimulation.

## Data Availability

The data recorded for this study will be made available upon request.
